# Sperm cryopreservation in Windsnyer boars; principles, technique, and updated outcomes

**DOI:** 10.1590/1984-3143-AR2022-0100

**Published:** 2023-10-09

**Authors:** Mamonene Angelinah Thema, Masindi Lottus Mphaphathi, Mahlatsana Ramaesela Ledwaba, Tshimangadzo Lucky Nedambale

**Affiliations:** 1 Animal Production, Germplasm Conservation and Reproductive Biotechnologies, Agricultural Research Council, Pretoria, South Africa; 2 Department of Animal Science, Tshwane University of Technology, Pretoria, South Africa

**Keywords:** glycerol, propanediol, ethylene glycol, cryopreservation, Windsnyer boars

## Abstract

The domestic pig breeds are in danger of extinction whereas the erosion of their gene pool is a serious concern because they significantly contribute to the rich biodiversity. Overall aim of this study was to determine the protocol for preserving the semen of the Windsnyer boars for conservation. A total of 18 ejaculates (6 replications/boar) were collected from three Windsnyer boars of proven fertility with the use of hand-gloved approach method, twice per week. Boars semen were pooled and extended with Beltsville Thawing Solution [(BTS) IMV Technologies, France], held at 18°C for 3 hours and centrifuged. The sperm pellet was re-suspended with Fraction A (20% egg yolk + BTS) and cooled at 5°C for 1 hour. Following cooling, semen was divided and diluted into different cryoprotectants (ethylene glycol, glycerol, propanediol, ethylene glycol + glycerol + propanediol) at equal contribution to make the total concentrations [4, 8, 12 and 16% and the 0% (control; without cryoprotectant)] and loaded into 0.25 mL straws. Two cryopreservation methods (liquid nitrogen vapour and controlled rated) were used to cryopreserve the semen straws. Semen straws were thawed at different temperatures (5, 18, 37 and 40°C) and evaluated for sperm motility, viability, and morphology traits. Post-thawed sperm total motility (36.0±5.3) and live normal sperm (49.5±8.3) percentages were recorded to be higher in the treatment supplemented with 16% glycerol (P<0.05). The highest sperm total motility percentage was recorded at 40°C (26.8±3.2) thawing temperature for liquid nitrogen vapour treatment (P<0.05). In conclusion, 16% glycerol was found to be the suitable cryoprotectant concentration for semen cryopreserved with liquid nitrogen vapour method and thawed at 40°C.

## Introduction

Windsnyer (wind-cutter) pig is a domestic breed with a short frame and narrower body, predominately found in South Africa, Mozambique, and Zimbabwe (Madzimure et al., 2012). This breed present characteristics not found in more productive and selected commercial breeds, such as adaptation to environmental conditions, good maternal qualities, longevity, and disease resistance which is a great interest in a world facing environmental challenges (Hlongwane et al., 2020). Their unique characteristics make them important genetic resources, which can be conserved (in or ex-situ) during this current era of climate change (Marsoner et al., 2018; Bovula et al., 2021). Therefore, population of indigenous pigs is, considered stagnated due to the absence of a comprehensive improvement and conservation strategy (Roberts and Lamberson, 2015).

The effective preservation of semen samples collected from Windsnyer boars may potentially enhance productivity, assist with biosecurity, and will allow for encouraging the international exchange of genetic material, and allow for sexing of sperm to facilitate (Parrilla et al., 2020). Liquid preservation is the most useful preservation method for boar semen, as it maintains higher sperm fertilizing ability as compared to cryopreservation (Vishwanath and Shannon, 2000; Watson, 2000). Cryopreservation was found to be successful in many mammalian species, whereas inefficient in porcine (Silva et al., 2015). This is due to the fact that boar sperm plasma membrane is different from that of the other animals; it consists of high poly-unsaturated fatty acid content and low concentrations of cholesterol (Yeste et al., 2017; Monteiro et al., 2022). As a result, cryopreservation process in boars may lead to changes of proteins and carbohydrate composition, which may disrupt membrane structure and reduce sperm viability (Silva et al., 2015).

The most common useful methods for cryopreserving boar semen are traditional (liquid nitrogen vapour) and slow freezing method (Kaeoket, 2012). Cryopreservation of boar semen has been studied for over a decade to develop protocols. As a results, semen extenders such as BTS and Androhep were tested as the protocol for preserving boar sperm in a liquid form at 15-18°C Martinez et al. (2001); the 3% glycerol, programmable freezer, and liquid nitrogen vapour were tested as the protocol for cryopreserving the boar sperm (Roca et al., 2003). The thawing temperature range is also one of the important factor that can affect the sperm survival rate. The correct temperature interval for thawing boar semen straw was reported to range between 36-70°C (Yeste et al., 2017). Additionally, a study by Jovičić et al. (2020) recorded the rapid boar semen thawing temperature of 40-70°C followed by a stabilizing procedure at 39°C to have maintained boar sperm quality traits.

Regardless of the protocols developed or tested, none of the protocols has shown to can maintain the boar sperm survival rate for an extended period. Hence only less than 1% of the cryopreserved boar sperm was used for artificial insemination and in vitro fertilization (Jovičić et al., 2020; Almubarak et al., 2021). Moreover, cryodamage induced during cryopreservation on the boar sperm, can be reduced by adding cryoprotectants or optimized cooling rates or lipoproteins to the sample (Wu et al., 2013).

Cryoprotectants can be classified as either penetrating or non-penetrating. Penetrating cryoprotectants (ethylene glycol, glycerol, and propanediol) are very effective in lowering the intracellular water cryopreservation point and intracellular ice crystal formation (Holt, 2000; Seshoka et al., 2016). Non penetrating cryoprotectants (avian egg yolk and lactose) increase the extracellular hyperosmotic gradient, dehydrate the cells, and limit the chance of intracellular ice crystal formation (Yánez-Ortiz et al., 2022). Furthermore, evaluations of different aspects of cryopreservation protocols demonstrated that the highest post-thawed motile or live boar sperm percentage could be achieved when using 2-4% glycerol (Jovičić et al., 2020). Whereas male variants within breeds differ in their ability to withstand freezing (Mapeka et al., 2009). Hence, researchers reported post-thawed sperm motility and viability of exotic breed to be different from the one of indigenous boars (Mapeka et al., 2009). The concentrations of cryoprotectant have been barely studied on boar semen. Furthermore, an exceeding 8 to 11% is the most used concentration in other species (Zong et al., 2022). Hence In the present study, the increase of cryoprotectant concentration from 3-16% were selected according to the unstable or unimproved results obtained in previous works. Therefore, a comparison of the commercially acceptable protocol for semen preservation would be in the best interest of the global porcine industry to improve the protocol. Preservation protocol will assist in improving reproduction, increasing the quality of pig production across the world and improve quality of the indigenous sperm conservation. The overall objectives of this study were to determine the suitable cryoprotectant (ethylene glycol, glycerol, and propanediol) concentrations (0, 4, 8, 12, and 16%) during cryopreservation of Windsnyer boars semen; to compare the cryopreservation methods (liquid nitrogen vapour and slow cryopreservation) and thawing temperatures (5, 18, 37, and 40°C) on post-thawed sperm motility, viability, and morphology of Windsnyer boars semen.

## Methods

### Experimental location and ethical clearance

The study was approved and carried out according to the guidelines of the Agricultural Research Council, Animal Production ethics committee [APAEC (2020/06)] and the Tshwane University of Technology Ethics Committee (AREC2021/10/009). The study was conducted at the Agricultural Research Council (ARC)-Irene at the Germplasm Conservation and Reproductive Biotechnology laboratory. The area is located at 25º 53´ 59.6ʺ South latitude and 28º 12´ 51.6ʺ East longitude in Pretoria, South Africa.

### Experimental boars

Three Windsnyer boars (>70 body weight) of the same age (13 months) were housed in an individual pen at the Pig Testing unit. They were fed with a daily ration of 2kg dry adult feed, per boar and had access to drinking water.

### Boars semen collection

Semen of the three Windsnyer boars of known fertility were sampled twice a week using the gloved-hand approach to get a total of 18 semen samples (6 replication). A thermo-flask filled with warm (37°C) water and a glass beaker covered with a gauze filter to separate the gel from the semen were used to collect the boars' sperm-rich fraction. Semen samples were taken to the lab for additional analysis within 30 minutes after collection.

### Preparation of the boars semen cryopreservation extenders

The cryopreservation extenders were prepared a day before use in 10mL tubes (Nest^®^, Biotechnology, China). The boars semen cryopreservation extenders were separated into two fractions (fraction A and fraction B). Fraction A extender consisted of BTS and 20% of chicken egg yolk while Fraction B consisted of 20% of chicken egg yolk, BTS, and cryoprotectant [ethylene glycol (Sigma-Aldrich®, United State of America), glycerol (Laboratory Consumables & Chemicals Supplies cc, South Africa), propanediol (Rochelle Chemicals & Lab Equipment, South Africa) and a combination of ethylene glycol + glycerol + propanediol] concentrations (0, 4, 8, 12 and 16%).

### Boars semen cryopreservation

Only sperm of greater than 70% motility and viability were pooled from the three individual Windsnyer boars for cryopreservation. Following pooling, semen was diluted with (1:2 v/v) BTS extender and evaluated for sperm motility, viability, and morphology. Diluted semen was then transferred to 15mL tubes (Nest^®^, Biotechnology, China) cooled at 18°C for 3 hours, and later was centrifuged at 800g 15°C for 10 minutes. Following centrifugation (Hettich Rotanta 460R, Tuttlingen, Germany), seminal plasma was discarded, and semen pellet was re-suspended (1:2) with Fraction A of extender and cooled at 5°C for 1 hour. Cooled semen was divided and diluted into different cryoprotectants (ethylene glycol, glycerol, propanediol, ethylene glycol + glycerol + propanediol) at equal contribution to make the total concentrations of 4, 8, 12, 16% and 0% (control; without cryoprotectant) and loaded into 0.25 mL straws at a final sperm concentration of 0.5 x 10^9^. Semen straws were equally allocated for liquid nitrogen vapour and slow (controlled rate) cryopreservation methods.

During the liquid nitrogen vapour method, semen straws were placed 3cm above liquid nitrogen level for 20 minutes in a polystyrene box. The semen straws were transferred immediately into the liquid nitrogen tanks following explore of vapour. During the controlled rate cryopreservation method, semen straws were transferred to the controlled rate freezer set at - 3°C/minute +5°C to -5°C; -30°C/minute from -5°C to -15°C and -50°C/minute from -15°C to -60°C. Furthermore, cryopreserved semen straws were plunged into the liquid nitrogen tank (-196°C) for preservation and further analysis. Thawing was accomplished by immersing the semen straws in warm (37°C) water for 1 minute or 40°C for 30 seconds or 18°C for 3 minutes or 5°C for 5 minutes followed by diluting the thawed content 1:1 in Beltsville Thawing Solution. Sperm motility, viability, and morphology percentages were then evaluated following dilution.

### Boars sperm motility evaluations

The sperm motility traits were evaluated using Sperm Class Analyzer^®^ (Microptin, Spain). Briefly, 5μL of diluted post-thawed or fresh semen was transferred to a warmed microscope glass slide (76x26x1mm-Wadmar-Knittel, Germany) and gently covered with a microscope cover slip. The sperm motility traits were then evaluated under 10x magnification with the Sperm Class Analyzer^®^ microscope projecting an image on a monitor. Two fields per treatment with an average number of 228 sperm and a final sperm concentration of approximately 32.7 × 10^6^/ml replication were captured at 10× magnification (Nikon^®^) during analysis (Thema et al., 2022).

### Boars sperm viability and morphology evaluations

The percentage of viable sperm and abnormalities (live with abnormal head, coiled tail, proximal and distal droplets sperm) were evaluated using Eosin-nigrosine staining (Onderstepoort, Faculty of Veterinary Sciences Pharmacy, South Africa). Briefly, 7μL of post-thawed or fresh semen was diluted in 20μL of Eosin-nigrosin staining. A 5μL of the diluted semen with the stain was smeared on a glass slide and stored at a room temperature to dry. The slides were then placed on a microscope (Olympus, BX 51FT, Tokyo, Japan) and a drop of immersion oil (Merck Chemicals, South Africa) was placed on the slide to evaluate sperm morphology and viability under 100x magnification. A total of 200 sperm per slide were evaluated and counted for each sample using a laboratory counter.

## Statistical analysis

The data were analysed with the use of two-way ANOVA tests to test the effect of cryoprotectants concentrations, cryopreservation methods and thawing temperatures on the sperm motility, viability and morphology traits. The trials were separated into two experiment: first experiment with the factorial design of 5x5 and the second experiment with the factorial design of 2x4. The trials were replicated 6 times per experiment. The F test was used to determine the significance difference. The results were expressed as mean values and standard deviation (SD). The p-value < 0.05 was considered statistically significant in the statistical analysis. All the statistical evaluations were performed using Statistical Product and Services Solutions (SPSS 11.5 for Windows, SPSS).

## Results

### To determine the suitable cryoprotectant concentrations during cryopreservation of Windsnyer boars semen

**Figure 1:**
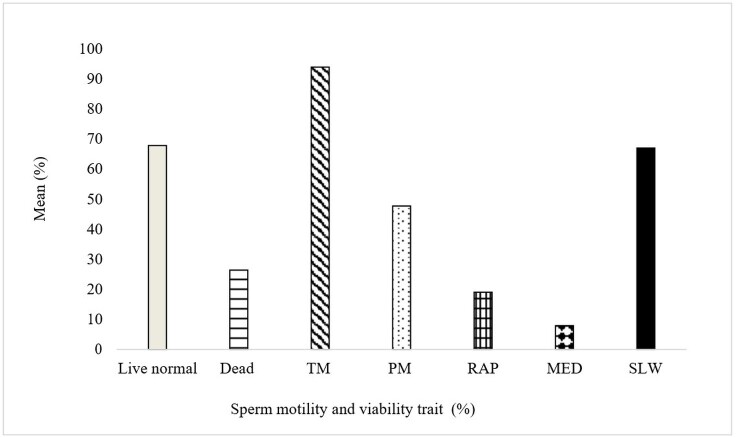
Sperm motility and viability traits of the fresh semen before preservation. TM: Total motility, PM: Progressive motility, RAP: Rapid motility, Med: Medium motility, SLW: Slow motility.

Greater than 70% of the sperm total motility and 65% of live normal sperm percentage were recorded in the fresh semen before preservation shown on Figure 1. Table 1 represent the post-thawed Windsnyer boars sperm viability and morphology traits cryopreserved with cryoprotectant concentrations. Post-thawed live normal sperm percentage was recorded higher in the treatment supplemented with 16% glycerol as compared to the other cryoprotectant concentrations (P<0.05). The lowest live normal sperm percentage was recorded in the semen cryopreserved with 16% ethylene glycol compared to the other cryoprotectant concentrations (P<0.05).

**Table 1:** Post-thawed Windsnyer boars sperm viability and morphology traits cryopreserved with cryoprotectant concentrations (Mean±SD).

**Treatment**	**CPA concentration (%)**	**Sperm viability and morphology trait**						
		**Live normal (%)**	**Dead** **(%)**	**Head abnormality (%)**		**Proximal droplet (%)**	**Distal droplet (%)**	**Coiled tail (%)**
CPA free	0	22.3±5.8^b^	77.7±6.4^a^		0±0^b^	0±0^b^	0±0^b^	0±0^b^
Glycerol	4	29.0±7.6^b^	71.0±7.6^b^		0±0^b^	0±0^b^	0±0^b^	0±0^b^
	8	23.0±9.3^c^	77.0±9.6^a^		0±0^b^	0±0^b^	0±0^b^	0±0^b^
	12	28.4±4.7^b^	71.0±6.9^b^		0.2±0.4^a^	0.2±0.4^a^	0.2±0.4^a^	0±0^b^
	16	17.0±6.6^c^	81.5±5.3^a^		0.5±0.5^a^	0.5±0.5^a^	0.5±0.5^a^	0±0^b^
Propanediol	4	32.1±6.7^b^	67.9±7.2^b^		0±0^b^	0±0^b^	0±0^b^	0±0^b^
	8	28.7±3.2^b^	71.3±3.3^b^		0±0^b^	0±0^b^	0±0^b^	0±0^b^
	12	29.0±3.5^b^	70.4±3.9^b^		0.3±0.5^b^	0.3±0.5^a^	0±0^b^	0±0^b^
	16	49.5±8.3^a^	50.5±7.5^c^		0±0^b^	0±0^b^	0±0^b^	0±0^b^
Ethylene glycol	4	25.7±9.5^b^	74.1±9.5^b^		0.2±0.4^a^	0±0^b^	0±0^b^	0±0^b^
	8	21.7±6.9^c^	78.1±6.9^a^		0.2±0.4^a^	0±0^b^	0±0^b^	0±0^b^
	12	35.5±9.5^b^	64.5±12.9^b^		0±0^b^	0±0^b^	0±0^b^	0±0^b^
	16	34.7±9.1^b^	65.3±9.1^b^		0±0^b^	0±0^b^	0±0^b^	0±0^b^
EG + GLY + PDO	4	28.8±4.4^b^	67.5±4.7^b^		0±0^b^	0.7±1.0^a^	0.7±0.8^a^	2.3±0.5^a^
	8	20.7±5.2^c^	78.5±5.1^a^		0±0^b^	0.5±0.5^a^	0±0^b^	2.0±2.2^a^
	12	21.8±3.5^c^	68.7±8.3^b^		0±0^b^	1.2±0.7^a^	0.7±1.0^a^	3.3±0.8^a^
	16	30.4±6.2^b^	65.5±6.1^b^		0±0^b^	0.9±0.8^a^	0.8±1.3^a^	2.3±1.4^a^

CPA: cryoprotectant. ^a-c^Values with different superscripts in a column differ significantly (P<0.05). EG: Ethylene glycol, GLY: Glycerol, PDO: Propanediol.

**Figure 2:**
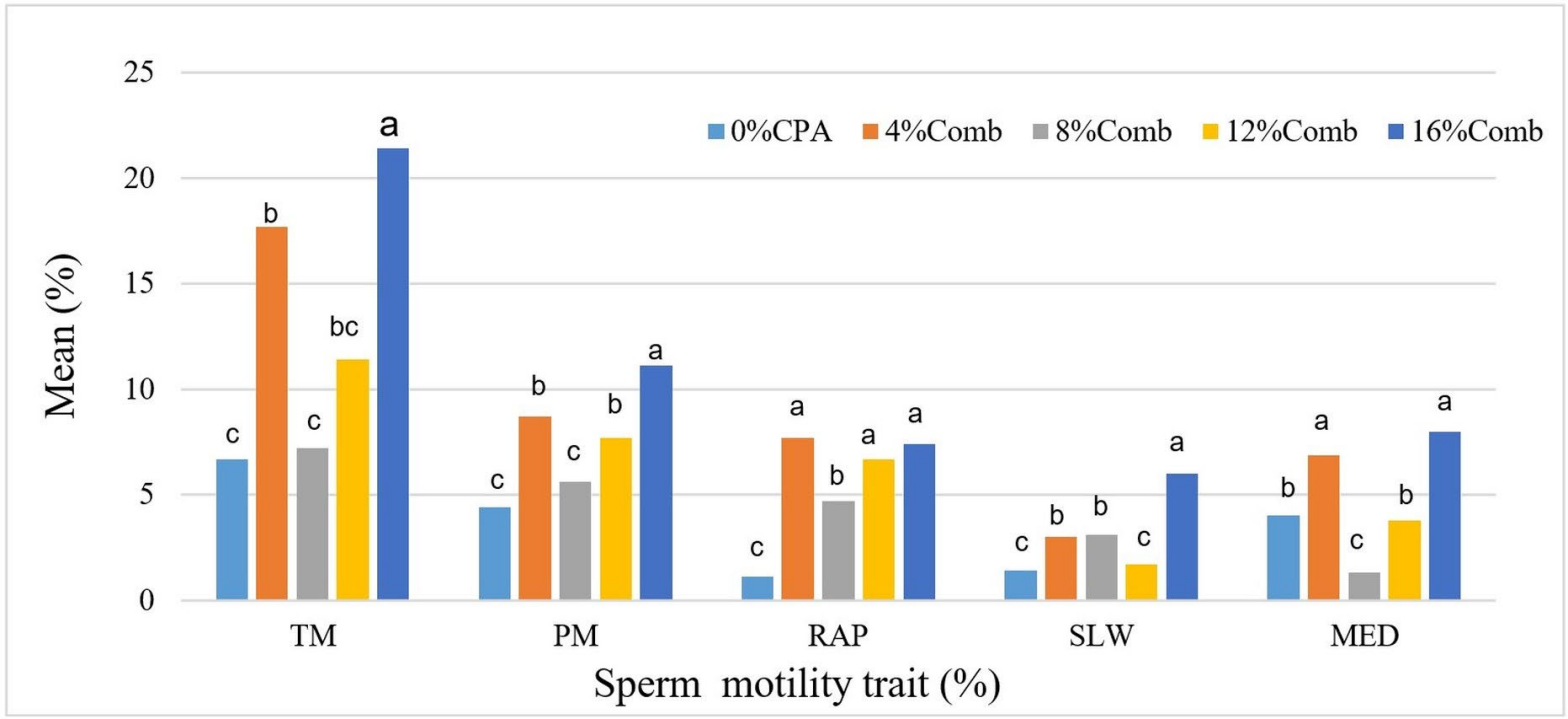
The effect of the combination of cryoprotectants concentrations on post-thawed Windsnyer boars sperm motility traits. ^a-c^Values with different superscripts differ significantly within the bars (P<0.05). Comb: combination; CPA: cryoprotectant; TM: total motility; PM: progressive motility; RAP: rapid motility; SLW: slow motility; MED: medium motility.

Post-thawed Windsnyer boars sperm motility traits cryopreserved with different cryoprotectant concentrations is represented on Table 2. There was no significant difference recorded on post-thawed sperm slow motility percentage irrespective of the different cryoprotectant concentrations (P<0.05). Figure 2 represents the effect of the combination of cryoprotectants concentrations on post-thawed Windsnyer boars sperm motility traits. The highest post-thawed sperm total motility percentage was recorded in the treatments supplemented with 16% of ethylene glycol + glycerol + propanediol (P<0.05).

**Table 2:** Post-thawed Windsnyer boars sperm motility traits cryopreserved with different cryoprotectant concentrations (Mean±SD).

**Sperm motility trait**	**CPA free**	**CPA**											
		**Ethylene glycol**				**Glycerol**				**Propanediol**			
	**0%**	**4%**	**8%**	**12%**	**16%**	**4%**	**8%**	**12%**	**16%**	**4%**	**8%**	**12%**	**16%**
TM (%)	8.0±1.8^b^	14.0±2.4^b^	9.0±3.9^b^	12.0±2.9^b^	9.0±4.6^b^	18.0±8.2^b^	19.0±6.2^b^	12.0±3.9^b^	36.0±5.3^a^	16.0±8.0^b^	6.0±2.7^b^	23.0±5.1^b^	19.0±8.1^b^
PM (%)	4.0±1.8^b^	8.0±3.1^b^	8.0±2.9^b^	8.0±4.5^b^	2.0±2.0^b^	8.0±6.4^b^	13.0±6.2^b^	8.0±5.2^b^	19.0±7.4^a^	8.0±3.2^b^	5.0±2.6^b^	16.0±8.4^a^	9.0±7.1^b^
RAP(%)	1.0±1.3^b^	4.0±4.1^b^	4.0±3.2^b^	5.0±6.1^b^	0.3±0.7^b^	4.0±4.1^b^	8.0±3.2^b^	2.0±2.2^b^	10.0±6.9^a^	3.0±2.6^b^	1.0±1.1^b^	9.0±9.0^b^	9.0±7.5^b^
MED(%)	4.7±1.8^b^	5.0±3.2^b^	5.0±4.2^b^	5.0±5.7^b^	5.7±2.5^b^	9.0±6.7^b^	7.8±3.4^b^	6.0±3.6^b^	19.0±11.7^a^	5.0±2.8^b^	4.0±3.5^b^	9.0±5.4^b^	5.0±4.6^b^
SLW (%)	2.3±1.7	5.0±2.8	0±0	2.0±1.9	3.0±2.7	5.0±4.8	3.2±2.4	4.0±5.2	7.0±6.1	8.0±5.2	1.0±1.1	5.0±5.4	5.0±2.7
STC (%)	92.0±1.9^a^	86.0±2.4^b^	91.0±3.9^ab^	88.0±2.9^ab^	91.0±4.5^ab^	82.0±8.2^b^	81.0±6.2^b^	86.0±3.9^b^	64.0±5.3^c^	84.0±8.0^b^	94.0±2.7^a^	77.0±5.1^c^	81.0±8.1^b^
BCF(Hz)	19.0±9.8^a^	13.0±4.8^b^	21.0±3.6^a^	21.0±9.2^a^	15.0±5.4^a^	14.0±6.1^b^	21.0±6.9^ab^	15.0±6.5^a^	16.0±5.7^a^	9.0±6.7^b^	18.0±3.6^a^	18.0±5.4^a^	12.0±9.5^b^

CPA: cryoprotectant; TM: total motility; PM: progressive motility; RAP: rapid motility; MED: medium motility; SLW: slow motility; STC: static; BCF: beat cross frequency. ^a-c^Values with a different superscript in a row differ significantly (P<0.05).

### To compare the cryopreservation methods and thawing temperatures on post-thawed sperm motility, viability, and morphology of Windsnyer boars semen

**Figure 3:**
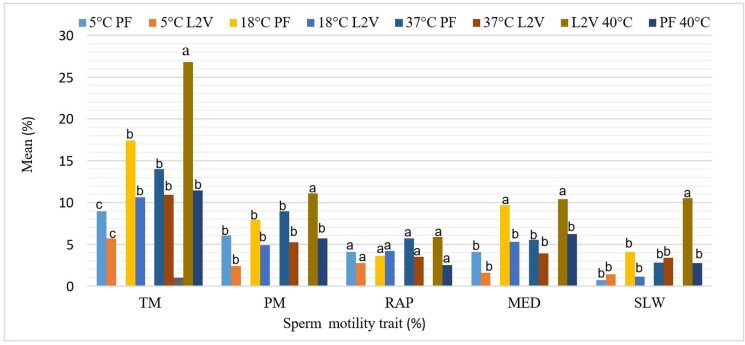
The effect of cryopreservation methods and thawing temperatures on the post-thawed Windsnyer boars sperm motility. ^a-c^Values with different superscripts differ significantly within the bars (P<0.05). TM: total motility; PM: progressive motility; RAP: rapid motility; SLW: slow motility; MED: medium motility; PF: programmable freezer; L2V: liquid nitrogen vapour.

The comparison of cryopreservation methods and thawing temperatures on Windsnyer boars sperm morphology and viability is presented on Table 3. Semen sample cryopreserved with liquid nitrogen vapour recorded the highest post-thawed live normal sperm percentage at a thawing temperature of 5°C, 18°C, 37°C and 40°C as compared to the controlled rate treatments (P<0.05). The effect of cryopreservation methods and thawing temperatures on the post-thawed Windsnyer boars sperm motility is described on Figure 3. The least post-thawed sperm total motility percentage was recorded on the treatments cryopreserved with controlled rated and liquid nitrogen vapour when thawed at 5°C, (P<0.05). Post-thawed sperm total motility for liquid nitrogen vapour and controlled rate freezer treatments showed no stability when the thawing temperatures were decreasing from 40-5°C (P<0.05).

**Table 3:** The comparison of cryopreservation methods and thawing temperatures on Windsnyer boars sperm morphology and viability (Mean±SD).

**Sperm viability and morphology trait**	**Cryopreservation method**							
	**Liquid nitrogen vapour**				**Slow freezing**			
	**5°C**	**18°C**	**37°C**	**40°C**	**5°C**	**18°C**	**37°C**	**40°C**
Live normal (%)	37.7±4.2^bc^	45.3±8.9^b^	44.2±8.9^b^	65.4±4.9^a^	26.5±6.6^c^	35.2±10.2^bc^	31.7±4.2^bc^	34.8±13.3^bc^
Dead (%)	60.8±14.2^b^	53.0±9.5^c^	52.5±9.5^c^	32.0±5.4^d^	73.5±6.2^a^	63.8±10.8^b^	65.3±5.5^b^	62.5±13.1^b^
Head abnormal (%)	0.5±0.3^b-d^	0.5±0.5^a^	1.0±0.9^a^	1.2±0.4^a^	0±0^b^	0.3±0.5^a^	0.8±0.9^a^	0.7±0.8^a^
Proximal droplet (%)	0.4±0.8^a^	0.5±0.5^a^	0.7±0.5^a^	0.3±0.5^a^	0±0^b^	0.3±0.5^a^	0.7±1.0^a^	0.8±0.8^a^
Distal droplets (%)	0.3±0.8^a^	0.5±0.5^a^	0.5±0.5^a^	0.3±0.5^a^	0±0^b^	0.2±0.5^a^	0.8±0.9^a^	0.6±0.8^a^
Coiled tail (%)	0.3±0.8^a^	0.2±0.4^a^	1.1±0.9^a^	0.8±0.4^a^	0±0^b^	0.2±0.4^a^	0.7±1.0^a^	0.6±0.8^a^

^a-d^Values with different superscripts in a row differ significantly (P<0.05).

## Discussion

### To determine the suitable cryoprotectant concentrations during cryopreservation of Windsnyer boars semen

The highest sperm motility and viability percentage were recorded in the treatment supplemented with 16% glycerol. Therefore, the successful increase of glycerol concentration (16%) as a cryoprotectant could be a novel suggestion for the improvement of cryopreservation techniques in boars semen. However, methodical evaluations of different aspects of cryopreservation protocol demonstrated that the highest post-thawed boars sperm motility and viability percentage was achieved when using 2-4% glycerol (Roca et al., 2003). However, the exceeding 8 to 11% is the most used concentration in other species (Zong et al., 2022). The post-thawed sperm motility and viability of exotic breed reported to be different from the one of indigenous boars (Mapeka et al., 2009).

The present study recorded glycerol to be the suitable cryoprotectant that maintained sperm motility, viability, and morphology more efficiently than ethylene glycol and propanediol. This may be due to the molecular weight of the glycerol (92.09g/mol) that makes it to be low toxic and to not allow rapid movement through the sperm (Morris et al., 2007). The present results agree with the results by Mapeka et al. (2009) which indicated glycerol as the suitable cryoprotectant for cryopreservation of Kolbroek (indigenous pig breed) boar semen; further, indicated that glycerol had yielded better results than other cryoprotectants such as Dimethyl sulfoxide.

Glycerol has been proven in many studies as the suitable cryoprotectant to cryopreserve boar semen. Furthermore, according to Yánez-Ortiz et al. (2022), different cryoprotectants have been tested but none has proven to be better at preserving boar sperm than glycerol. However according to Seshoka et al. (2016), ethylene glycol has a higher penetrating ability into the cell, which may cause damage to the membrane; further, added that ethylene glycol cannot be associated with glycerol while it has a lower molecular weight (62.07g/mol). Whereas propanediol is the common cryoprotectant used to freeze the bovine oocytes with a molecular weight of 76.09g/mol.

The previous studies reported boar semen cryopreservation to significantly decrease more than 70-80% sperm viability. Latest studies showed that greater than 70% of boar sperm do not survive cryopreservation (Caamaño et al., 2021; Bustani and Baiee, 2021). Additionally, farrowing rates decreased by 20-30% and litter sizes dropped by two to three piglets when using cryopreserved boar semen in comparison to rates attained when using liquid preservation (Kaeoket et al., 2008). This is due to the crystal formation occurring during cryopreservation, followed by dehydration that ends up affecting the survival rate of the boar sperm (Almubarak et al., 2021). There was no cryoprotectant effect shown when ethylene glycol + glycerol + propanediol were combined at different concentrations on boars sperm quality. To our knowledge, this was the first study to have combined ethylene glycol + glycerol + propanediol at different concentrations on boars semen. In the previous studies, glycerol has been combined with Dimethylacetamide or Dimethyl sulfoxide (Mapeka et al., 2009). As a result, glycerol alone or combined had maintained the boars sperm quality.

### To compare the cryopreservation methods and thawing temperatures on post-thawed sperm motility, viability, and morphology of Windsnyer boars semen

The present study recorded liquid nitrogen vapour as the suitable cryopreservation method to have maintained the Windsnyer boars sperm quality. The values of the live sperm percentages for different thawing temperature treatments were higher in the semen cryopreserved with the aid of liquid nitrogen vapour. Which agrees with the study by Kaeoket (2012) documented that, the most common useful cryopreservation methods for boar semen cryopreservation were traditional (liquid nitrogen vapour) and controlled rate (slow freezing). The present results recorded the highest significant reduction of sperm motility and viability percentages in the treatments cryopreserved with a controlled rate method. This is because controlled rate freezer (slow freezing) is associated with temperature changes when cooling rates changes from 5 to -160°C which invites the risk of intracellular ice crystal formation, osmotic changes and sperm shrinkage or injury and that led to a decrease in sperm survival (Yánez-Ortiz et al., 2022). This agrees with the study by Sancho et al. (2007), which indicated that the temperature below 5°C imposed the greatest effect during cryopreservation of the boar semen. Additionally, Yeste et al. (2017) indicated that boar post-thawed sperm survival ranged between 25-60%.

The present results indicated the sperm cryopreserved with the aid of liquid nitrogen vapour and thawed at 40°C to have maintained the sperm motility and viability quality. This is due to that, high thawing temperatures enable quick passage through the dangerous re-crystallization phase (50 to 0°C) and it forces ice crystal formation to change from a glassy to a liquid in order to prevents cellular damage (Tomás-Almenar and Mercado, 2022). A study by Jovičić et al. (2020) showed the increasing thawing boar semen temperature of 70°C for 8 seconds followed by a stabilizing procedure of 39°C for 52 seconds to have proven to can maintain the boar sperm quality traits. Moreover, several research reported that increase in the temperature during thawing enhanced post-thawed motility (Salih et al., 2021; Tomás-Almenar and Mercado, 2022).

## Conclusions

The combination of glycerol, ethylene glycol, and propanediol did not maintain sperm motility or viability after the freezing-thawing process when compared to the 16% glycerol treatment group. As a result, 16% glycerol is suggested as the optimal concentration for Windsnyer boar sperm freezing using the liquid nitrogen vapor method and thawing temperature of 40°C.
